# Sedation-related complications during anesthesiologist-administered sedation for endoscopic retrograde cholangiopancreatography: a prospective study

**DOI:** 10.1186/s12871-020-01048-0

**Published:** 2020-05-28

**Authors:** Chengcheng C. Zhang, Nicole Ganion, Phillip Knebel, Christian Bopp, Thorsten Brenner, Markus A. Weigand, Peter Sauer, Anja Schaible

**Affiliations:** 1 Department of Gastroenterology, Heidelberg University Hospital, Im Neuenheimer Feld 410, 69120 Heidelberg, Germany; 2 Department of Anesthesiology, Heidelberg University Hospital, Heidelberg, Germany; 3 Department of General Surgery, Heidelberg University Hospital, Heidelberg, Germany; 4 Department of Anesthesiology, GRN Hospital Schwetzingen, Schwetzingen, Germany

**Keywords:** Anesthesia, Hypotension, Hypoxemia, ERCP, Endoscopic retrograde cholangiopancreatography, Sedation

## Abstract

**Background:**

Patients undergoing endoscopic retrograde cholangiopancreatography (ERCP) require adequate sedation or general anesthesia. To date, there is lack of consensus regarding who should administer sedation in these patients. Several studies have investigated the safety and efficacy of non-anesthesiologist-administered sedation for ERCP; however, data regarding anesthesiologist-administered sedation remain limited. This prospective single-center study investigated the safety and efficacy of anesthesiologist-administered sedation and the rate of successful performed ERCP procedures.

**Methods:**

The study included 200 patients who underwent ERCP following anesthesiologist-administered sedation with propofol and remifentanil. Procedural data, oxygen saturation, systolic blood pressure (SBP), heart rate, recovery score, patient and endoscopist satisfaction, as well as 30-day mortality and morbidity data were analyzed.

**Results:**

Sedation-related complications occurred in 36 of 200 patients (18%) and included hypotension (SBP < 90 mmHg) and hypoxemia (O_2_ saturation < 90%) in 18 patients (9%) each. Most events were minor and did not necessitate discontinuation of the procedure. However, ERCP was terminated in 2 patients (1%) secondary to sedation-related complications. Successful cannulation was performed in all patients. The mean duration of the examination was 25 ± 16 min. Mean recovery time was 14 ± 10 min, and high post-procedural satisfaction was observed in both, patients (mean visual analogue scale [VAS] 9.6 ± 0.8) and endoscopists (mean VAS 9.3 ± 1.3).

**Conclusion:**

This study suggests that anesthesiologist-administered sedation is safe in patients undergoing ERCP and is associated with a high rate of successful ERCP, shorter procedure time, and more rapid post-anesthesia recovery, with high patient and endoscopist satisfaction.

## Background

Endoscopic retrograde cholangiopancreatography (ERCP) is a complex and time-consuming procedure necessitating adequate sedation or general anesthesia. Reportedly, complication and mortality rates associated with ERCP are 5–10% and 0.1–1%, respectively [1–5]. Complications of ERCP include acute pancreatitis, bleeding, and perforation [1].

To date, optimal sedation techniques for complex endoscopic procedures remain unclear. There is lack of global consensus regarding the choice of practitioners to administer sedation and the optimal sedation technique for ERCP. In some countries (e.g., France), sedation is performed only by anesthesiologists. However, the German ‘Update S3-guideline: sedation for gastrointestinal endoscopy 2014’ clearly defines and summarizes the staff and technical requirements as follows [6]: Sedation can be administered by a trained nurse under a physician’s supervision during simple endoscopic examinations. A second physician with experience in intensive care medicine should be present in cases involving a high procedural risk and for those requiring prolonged complex endoscopic interventions. Anesthesiologist-administered sedation is necessary in high-risk patients (those categorized as American Society of Anesthesiologists [ASA] class III–IV, in those undergoing difficult endoscopic interventions or in those with complex anatomy predisposing to a high risk of airway obstruction).

All sedation techniques are associated with the risk of cardiopulmonary complications, such as hypoventilation, respiratory depression, apnea, hypotension, and bradycardia [7]. A Cochrane Review evaluated the efficacy and safety of sedation techniques for ERCP in adults [7]. The authors intended to compare complication rates between sedation performed by anesthesia- and non-anesthesia personnel. However, they could not identify relevant studies involving anesthesia personnel. Therefore, the authors analyzed the results of 4 randomized trials that compared midazolam and meperidine with propofol-only sedation in patients undergoing ERCP with sedation performed by non-anesthesia personnel. No significant differences were observed in cardiorespiratory complications, and no immediate mortality was reported. Patients receiving propofol-only sedation for ERCP showed more rapid post-anesthesia recovery than patients receiving midazolam and meperidine [7–11], and the former group also showed higher patient satisfaction [7, 9, 10].

In all studies identified by the Cochrane Review, sedation was performed only by non-anesthesia personnel. Therefore, whether anesthesiologist-administered sedation affects sedation-related complications is still elusive [7]. To date, few studies have described ERCP using sedation administered by anesthesiologists. The role of anesthesia personnel in the administration of sedation and the effects of sedation administered by anesthesia personnel with regard to the safety profile and complication rates of sedation during complex endoscopic procedures remains unclear. This prospective cohort study investigated the efficacy and safety of anesthesiologist-administered sedation for ERCP.

## Methods

### Study design

Our study protocol conformed to the Declaration of Helsinki and was approved by the Institutional Ethics Committee vide letter no S-457/2013. Written informed consent was obtained from all patients included in the study.

This prospective single-center study was performed at the Interdisciplinary Endoscopy Center, University Hospital of Heidelberg and included all adults who underwent ERCP at this center between March 2014 and November 2014. During this study period, all ERCP procedures were performed using anesthesiologist-administered sedation with only propofol and remifentanil. Baseline patient characteristics (age, sex, height and weight) were recorded. ERCP was performed by three experienced specialists in interventional endoscopy.

### Study population

Exclusion criteria were age < 18 years, pregnancy, lack of informed consent (e.g. patients with mental retardation or language issues), a history of propofol and/or remifentanil allergy, baseline O_2_ saturation (O_2_ sat) < 90%, baseline systolic blood pressure (SBP) < 90 mmHg, and need for general anesthesia.

### Data collection

Following evaluation, patients were categorized based on the ASA physical status classification. Patient monitoring during ERCP included clinical observation, non-invasive blood pressure measurement every 5 min, continuous monitoring of O_2_ sat, heart rate and electrocardiography. Patient characteristics, cardiorespiratory and procedural data, sedation-related events, and patient and endoscopist satisfaction data were recorded in a case report format before, during, and after examination.

All patients were transferred to a recovery unit and monitored by a nurse after ERCP. Patients were evaluated 30 min after termination of administration of sedation for post-anesthesia recovery using the “Post Anesthesia Recovery Score” (PARS), which evaluates patients’ status with regard to the following criteria: *activity* (able to move all 4 extremities voluntarily or on command [2 points], able to move 2 extremities voluntarily or on command [1 point], unable to move extremities voluntarily or on command [0 points]), *consciousness* (fully awake [2 points], arousable on calling [1 point], unresponsive [0 points]), *circulation* (SBP ± 20% of pre-anesthetic level [2 points], SBP ± 20–49% of pre-anesthetic level [1 point], SBP ± 50% of pre-anesthetic level [0 points]), *respiration* (able to breathe deeply and cough freely [2 points], dyspnea or limited breathing [1 point], apneic [0 points]), and *color* (normal [2 points], pale, dusky, blotchy, jaundiced, or other [1 point], cyanotic [0 points]) [12]. Complete recovery was defined as a maximum score of 10 points. Patients were discharged from the recovery unit after fully recovered. Furthermore, after regaining full consciousness, all patients filled a questionnaire and a 6-point Likert scale (1: very satisfied, and 6: very dissatisfied) regarding patient satisfaction and readiness to undergo a repeat examination under the same conditions. Endoscopist satisfaction was also recorded using a 6-point Likert scale.

After the procedure, patients were followed-up for 30 days to assess complications, as well as morbidity and mortality. Post-ERCP pancreatitis was defined as acute onset of epigastric pain, elevated serum lipase/amylase levels (at least 3-fold higher than the upper limit of normal), and characteristic imaging findings. Patients with at least 2 of these 3 criteria were diagnosed with post-ERCP pancreatitis. According to the Atlanta classification system, severity of pancreatitis was categorized as mild pancreatitis (absence of organ failure and local or systemic complications), moderate pancreatitis (transient organ failure resolving within 48 h and/or local or systemic complications without persistent organ failure), and severe pancreatitis (organ failure persisting > 48 h) [13, 14].

### Sedation techniques

All patients received continuous oxygen supplementation during the procedure at the rate of 4 l/min via nasal cannula. Xylocaine spray (3 jets) was used for oropharyngeal anesthesia without any other oral premedication. According to the German ‘Update S3-guideline: sedation for gastrointestinal endoscopy 2014’ an initial propofol loading dose of 40 – 60 mg (depending on age, body weight and comorbidities of the patient) was administered through an intravenous catheter for sedation followed by body-weight adapted continuous infusion of propofol (1.5–4.5 mg/kg/hour) and remifentanil (0.025–0.2 μg/kg/min) [6]. An additional propofol bolus (10 – 20 mg) was injected in patients showing signs of discomfort (e.g. agitation, uncontrolled movements, facial expressions and sounds).

### Primary and secondary endpoints

Primary endpoints of this study included sedation-related complications, i.e., hypoxemic events (defined as desaturation represented by O_2_ sat < 90% for at least 2 min), hypotension (defined as SBP < 90 mmHg), bradycardia (defined as heart rate < 40 beats per min [bpm]), failure to complete ERCP secondary to sedation-related complications, 30-day mortality secondary to sedation, anesthesia or ERCP-related complications. Secondary endpoints included endoscopist and patient satisfaction, patients’ willingness to undergo a repeat examination under the same conditions, deep cannulation rate, and time until intubation (defined as time between insertion of the endoscope through the pharynx and intubation of the major papilla), rate of successful ERCP, duration of the procedure (defined as time between insertion of the endoscope through the pharynx and removal of the endoscope from the pharynx), cumulative doses of propofol (mg) and remifentanil (μg), time until recovery after sedation (defined as time between termination of the procedure and transfer to recovery unit).

### Statistical analysis

Descriptive statistics were used for all parameters. Results are expressed as means ± standard deviation and ranges for continuous variables and numbers and percentages for categorical variables. Values recorded for the 6-point Likert-scale (1: very satisfied and 6: very dissatisfied) were converted into a 10-point visual analogue scale (VAS) (0: very dissatisfied and 10: very satisfied) by linear transformation. All data were analyzed using the IBM SPSS Statistics 24 software.

## Results

**Fig. 1:**
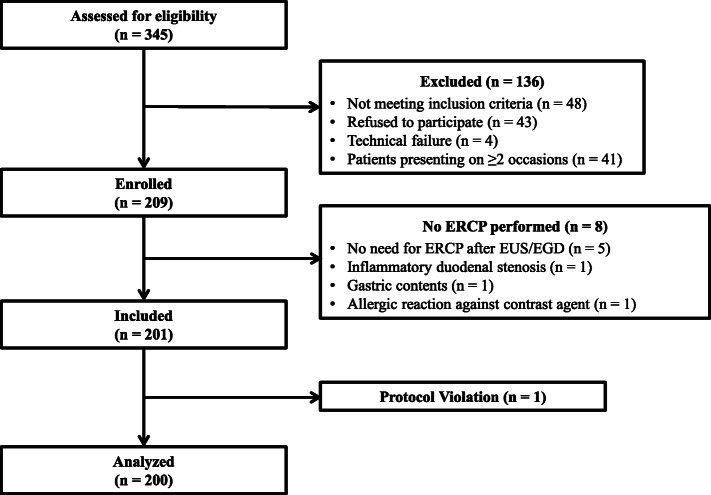
Study flow chart, EGD: esophagogastroduodenoscopy, ERCP: endoscopic retrograde cholangiopancreatography, EUS: endoscopic ultrasound

During the study period, 345 patients underwent ERCP and were assessed for eligibility to be included in the study (Fig. 1). As shown in Fig. 1 and Table 1, 136 patients could not be enrolled owing to the following reasons: 43 patients refused to participate, 48 did not meet the inclusion criteria (*n* = 7: baseline O_2_ sat < 90%, *n* = 3: basal SBP < 90 mmHg, *n* = 30: lack of written informed consent for various reasons, *n* = 2: age < 18 years, *n* = 6: need for general anesthesia), technical failure occurred in 4 patients, and 41 patients were excluded because they presented for ERCP on ≥2 occasions during the study period. In these cases, patients’ data were included in the statistical analysis only once. Eventually, 209 patients were enrolled. However, 8 patients were secondarily excluded because ERCP could not be performed owing to non-sedation related causes (7 patients were excluded because they underwent endoscopic ultrasonography or gastroscopy instead of ERCP, and 1 patient was excluded owing to an allergic reaction against the contrast agent administered during the ERCP). Therefore, 201 patients were included in the study. Notably, 1 patient who accidentally received additional drugs (different from the prescribed protocol) for sedation was excluded. After exclusion of patients owing to the aforementioned reasons, 200 patients were investigated.

**Table 1:** Characteristics of patients excluded from the study

Excluded patients (not meeting inclusion criteria)	n
	**48**
O_2_ sat < 90%	7
SBP < 90 mmHg	3
Lack of written informed consent	30
Age < 18 years	2
Need for general anesthesia	6
**Patients who refused to participate**	**43**
**Technical failure**^**a**^	**4**
**Patients presenting on ≥ 2 occasions**^**b**^	**41**
**No ERCP performed**	**8**
No need for ERCP after EUS/EGD	5
Inflammatory duodenal stenosis	1
Gastric contents	1
Allergic reaction against the contrast agent	1
**Protocol violation**^**c**^	**1**
**Total**	**Σ 145**

*EGD* Esophagogastroduodenoscopy, *ERCP* Endoscopic retrograde cholangiopancreatography, *EUS* Endoscopic ultrasound, *O*_*2*_*sat* Oxygen saturation, *SBP* Systolic blood pressure

^a^Technical failure refers to monitoring system failure such that vital parameters could not be completely and accurately recorded

^b^Patients who presented on ≥2 occasions were included in the study only once. The most complete data set was chosen for evaluation. In cases of data sets that were identical with regard to recorded data, data were chosen randomly

^c^Protocol violation was observed in 1 patient who was accidentally administered additional drugs (different from those included in the study protocol) for sedation

Table 2 shows the baseline clinical and demographic characteristics of the investigated patients. Notably, the study included 64% men (*n* = 128/200), and the age of the study population was 19–89 years (mean 56.3 years). Most patients were classified as ASA class III (59.5%, *n* = 119/200) and class II (36.5%, *n* = 73/200). The most common underlying disease was a hepatic disorder in 63.5% of the patients (*n* = 127/200), and the most common indication for ERCP was malignant biliary stenosis in 58/200 patients (29%), followed by postoperative stricture at the biliary anastomosis after liver transplantation in 22.5% of the patients (*n* = 45/200). The mean total propofol dose administered for sedation was 287 ± 134 mg (range 80–800 mg) with a medication dosage of 3.8 ± 1.7 mg/kg (range 1–10.3 mg/kg). The mean total remifentanil dose administered was 135 ± 68 μg (range 20–500 μg) with a medication dosage of 1.8 ± 1.0 μg/kg (range 0.3–6.4 μg/kg).

**Table 2:** Patients’ clinical and demographic characteristics, procedural indications, and medication dosages

Male, n (%)	128/200 (64)
Mean age ± SD, (years)	56.3 ± 15.2
**ASA score, n (%)**	
I	7 (3.5)
II	73 (36.5)
III	119 (59.5)
IV	1 (0.5)
**Disorder, n (%)**	
Hepatic	127/200 (63.5)
Gastrointestinal	31/200 (15.5)
Postoperative	20/200 (10)
Other disorders	22/200 (11)
**Procedural indications, n (%)**	
Malignant jaundice	58/200 (29)
Stricture after LTX	45/200 (22.5)
Stones/sludge	37/200 (18.5)
Postoperative complications	18/200 (9)
Other reasons	42/200 (21)
**Medication dosages**	
Propofol mean dose ± SD, (mg)	287 ± 134
Propofol mean dosage ± SD, (mg/kg)	3.8 ± 1.7
Remifentanil mean dose ± SD, (μg)	135 ± 68
Remifentanil mean dosage ± SD, (μg/kg)	1.8 ± 1.0

ASA I: normal healthy patients, ASA II: patients with mild systemic disease, ASA III: patients with severe systemic disease, ASA IV: patients with severe systemic and life-threatening disease

*ASA* American Society of Anesthesiologists

*LTX* Liver transplantation, *SD* Standard deviation

### Cardiorespiratory data

Cardiorespiratory parameters are presented in Table 3. We observed baseline O_2_ sat of 98.0 ± 1.6%. Desaturation (represented by O_2_ sat < 90%) was recorded in 18 of 200 patients (9%) during ERCP. Of these 18 patients, hypoxemia was recorded once in 11 patients (61%) and twice in 5 patients (28%) during the entire period of investigation. We observed 3 episodes of hypoxemia and 4 episodes of desaturation in 1 patient each (5.5%) during the entire procedure. The mean O_2_ sat was 97.7 ± 2.7% (range 65–100%). Most hypoxemic complications were minor events, and patients were successfully treated with minor airway interventions (e.g., chin lift, jaw thrust, and/or increased oxygen supplementation via nasal cannula) and did not necessitate discontinuation of ERCP. ERCP was terminated in 2 patients secondary to sedation-related hypoxemia: 1 patient showed oxygen saturation decrease secondary to aspiration. However, O_2_ sat was rapidly restored to safe levels after termination of the ERCP by placing the patient in the supine position with assisted ventilation support via a respiratory mask. Spontaneous respiration was restored, and the patient was awake when transferred to the recovery unit with an O_2_ sat of 99% on 2 l of oxygen supplementation via nasal cannula. ERCP was repeated 2 days later and was successfully performed under general anesthesia. ERCP was terminated in the second patient owing to apnea with recurrent decline in O_2_ sat to < 90% and agitation. O_2_ sat improved following increased oxygen supplementation via nasal cannula, and after recovery from sedation, spontaneous respiration was restored with an O_2_ sat of 99% without oxygen supplementation when the patient arrived at the recovery unit. The patient recovered completely from this sedation-related complication. ERCP was not repeated in this case because cholestasis resolved after the initial ERCP.

**Table 3:** Patients’ cardiorespiratory data

**O**_**2**_**sat**	
Baseline O_2_ sat ± SD (%)	98.0 ± 1.6
O_2_ sat < 90%, n (%)	18/200 (9)
*n* = 1, n (%)	11/18 (61)
*n* = 2, n (%)	5/18 (28)
*n* = 3, n (%)	1/18 (5.5)
*n* = 4, n (%)	1/18 (5.5)
Apnea, n (%)	1/200 (0.5)
Mean O_2_ sat ± SD (range), (%)	97.7 ± 2.7 (65─100)
**SBP**	
SBP baseline ± SD, (mmHg)	142 ± 21
Mean SBP ± SD (range), (mmHg)	128 ± 24 (74─220)
SBP < 90 mmHg, n (%)	18/200 (9)
*n* = 1, n (%)	7/18 (39)
*n* = 2, n (%)	7/18 (39)
*n* = 3, n (%)	2/18 (11)
*n* = 4, n (%)	2/18 (11)
**HR**	
Baseline HR ± SD, (bpm)	79 ± 14
Mean HR ± SD (range), (bpm)	80 ± 15 (40─128)
Bradycardia, n (%)	0/200 (0)
**Termination of procedure secondary to adverse events, n (%)**	**2/200 (1)**

*HR* Heart rate, *O*_*2*_*sat* Oxygen saturation, *SBP* Systolic blood pressure, *SD* Standard deviation

The baseline SBP was 142 ± 21 mmHg. Hypotension (SBP < 90 mmHg) occurred in 18 of 200 patients (9%). Among these 18 patients, a single episode of hypotension was recorded in 7 patients (39%) and 2 episodes of hypotension in 7 patients (39%) during the entire period of investigation. More than 2 episodes of hypotension occurred in 4 patients during the entire procedure (3 episodes: *n* = 2/18, 4 episodes: *n* = 2/18). The mean SBP was 128 ± 24 mmHg (range 74 mmHg–220 mmHg). Hypotension was successfully treated with intravenous fluid replacement in all patients, and no patient needed adrenergic drug administration. All procedures could be continued until completion, and all patients with hypotension recovered completely.

The mean heart rate was 79 ± 14 bpm (range 40–128 bpm). No patient developed bradycardia (heart rate < 40 bpm) during the investigation.

Sedation-related complications (hypotension and hypoxemia) occurred in 36 of 200 patients (18%), and ERCP was terminated in 2 patients (1%) secondary to sedation-related hypoxemia.

### Cannulation rate and procedural data

As shown in Table 4, cannulation of the major duodenal papilla was performed successfully in all patients (*n* = 200, 100%). The mean duration of ERCP was 25 ± 16 min, with a maximum and minimum duration of 85 and 3 min, respectively. The mean time for intubation of the major papilla was 7.00 ± 6.5 min (range 1 min–53 min).

**Table 4:** Cannulation rate, procedural data and satisfaction in patients and endoscopists

**Cannulation**	
Successful cannulation, n (%)	200 (100)
Mean time for intubation±SD, (min)	7 ± 6.5
Range, (min)	1 ─ 53
**Procedural data**	
Mean duration±SD, (min)	25 ± 16
Range, (min)	3 ─ 85
**Endoscopist-reported satisfaction**	
Mean VAS ± SD	9.3 ± 1.3
**Patient-reported satisfaction**	
Mean VAS ± SD	9.6 ± 0.8
Willingness to undergo a repeat procedure under the same conditions, n (%)	197/200 (98.5)

*SD* Standard deviation, *VAS* Visual analogue scale

### Post-anesthesia recovery data

Post-anesthesia recovery data are shown in Table 5. We observed that 97% of the patients were awake and oriented upon arrival at the recovery unit (*n* = 194/200), and the mean recovery time after sedation was 14 ± 10 min. Based on the PARS tool, post-anesthesia recovery was assessed 30 min after completion of ERCP. As shown in Table 5, 60% of the patients (*n* = 120/200) recovered completely with a maximum PARS value achieved 30 min after the investigation. The mean PARS value was 9.5 ± 0.7 (minimum of 7 to a maximum of 10 points).

**Table 5:** Post-anesthesia recovery data

Post-anesthesia recovery data	
PARS after 30 min, n (%)	
10	120/200 (60)
9	60/200 (30)
8	15/200 (7.5)
7	5/200 (2.5)
Mean PARS±SD	9.5 ± 0.7
Recovery time ± SD, (min)	14 ± 10
Patients awake/oriented upon arrival at the recovery unit, n (%)	194/200 (97)
**Activity, n (%)**	
Able to move 4 extremities voluntarily or on command	186/200 (93)
Able to move 2 extremities voluntarily or on command	14/200 (7)
Unable to move extremities voluntarily or on command	0/198 (0)
**Respiration, n (%)**	
Able to breathe deeply and cough freely	200/200 (100)
Dyspnea or limited breathing	0/200 (0)
Apneic	0/200 (0)
**Circulation, n (%)**	
SBP ± 20% of pre-anesthetic level	131/196 (67)
SBP ± 20─49% of pre-anesthetic level	61/196 (31)
SBP ± 50% of pre-anesthetic level	4/196 (2)
**Consciousness, n (%)**	
Fully awake	189/200 (94.5)
Arousable on calling	11/200 (5.5)
Unresponsive	0/200 (0)
**Color, n (%)**	
Normal	199/200 (99.5)
Pale, dusky, blotchy, jaundiced, or other	1/200 (0.5)
Cyanotic	0/200 (0)

*PARS* Post Anesthesia Recovery Score, *SBP* Systolic blood pressure, *SD* Standard deviation

All patients were able to breathe deeply and cough freely (*n* = 200/200, 100%). Regarding circulatory status, data were unavailable in 4 patients (for various reasons). SBP ± 20% of the pre-anesthetic level was observed in 67% of the patients (*n* = 131/196), and SBP ± 20–49% of the pre-anesthetic level was observed in 31% of the patients (*n* = 61/196). 94.5% of the patients were completely awake after 30 min (*n* = 189/200). Complete resumption of activity was observed in 186 of 200 patients (93%), and normal skin color was restored in 99.5% of the patients (*n* = 199/200).

### Patient and endoscopist satisfaction

As shown in Table 4, the mean VAS of the endoscopy team with regard to sedation quality was significantly high (mean VAS: 9.3 ± 1.3). Patient satisfaction scores with regard to sedation quality were also high (mean VAS: 9.6 ± 0.8), and 197 of 200 patients were willing to undergo a repeat procedure under the same conditions (98.5%).

### Mortality and morbidity data

No sedation-related complications occurred during the 30 days follow-up period. Post-ERCP complications within 30 days occurred in 9 of 200 patients (4.5%). Major complications were cholangitis, which occurred in 3 of 200 patients (1.5%) and pain (*n* = 3/200, 1.5%), followed by post-ERCP pancreatitis (*n* = 2/200, 1%) and bleeding (*n* = 1/200, 0.5%). Both patients (1 with mild and the other with moderate pancreatitis according to the Atlanta classification system) who developed post-ERCP pancreatitis recovered without any complications after standard therapeutic interventions.

The 30-day mortality rate after ERCP observed in this study was 1%. Two patients died within 30 days of non-sedation-related causes: 1 patient developed acute coronary heart syndrome after surgery and the other died of cancer.

## Discussion

To date, optimal sedation techniques for complex endoscopic procedures are controversial. Additionally, the safety and efficacy of anesthesiologist-administered sedation vs. sedation administered by non-anesthesia personnel remain unclear. Our large-scale prospective cohort study investigated the safety and efficacy of anesthesiologist-administered sedation for ERCP.

Results of non-anesthesiologist administered sedation of studies included in the Cochrane Review [7–11], and results of anesthesiologist-administered sedation of the present study are shown in Table 6. The mean medication dose of propofol used in our study was lower than that used in studies included in the Cochrane Review, which described sedation administered by non-anesthesia personnel [7–11]. However, Goudra et al. [15] reported a lower mean propofol dosage in cases of sedation administered by non-anesthesia personnel, which however was associated with significantly low patient and endoscopist satisfaction. Reportedly, the quality of sedation administered by anesthesia personnel was good, although compared with our study, their study required significantly higher propofol doses [15]. In our study, propofol (a sedative without analgesic properties) complemented the action of remifentanil (an opioid analgesic) against ERCP-associated discomfort. The lower propofol doses used in our study are attributable to the synergistic anesthetic effect of these drugs administered to all patients [16].

**Table 6:** Results of non-anesthesiologist administered sedation of previous studies and results of anesthesiologist-administered sedation of present study

**Studies**	**Number of patients**	**Age, years**	**Gender, [m/f]**	**ASA**	**Procedure**	**Mean propofol [mg]**
Vargo et al. [11]	38	52.9 ± 2.4	21 / 17	ASA I: 14 (36.8%) ASA II: 16 (42.1%)ASA III: 8 (21.1%) ASA IV: -	EUS ERCP	356.8
Riphaus et al. [9]	77	83.7 ± 7.8	35 / 42	ASA I: - ASA II: - ASA III: 32 (41.6%) ASA IV: 39 (50.6%)	ERCP	322
Kongkam et al. [8]	67	52.3 ± 11.9	40 / 27	ASA I: 26 (38.8%) ASA II: 22 (32.8%) ASA III: 19 (28.4%) ASA IV: -	ERCP	299.9
Schilling et al. [10]	76	82.4	25 / 51	ASA I: - ASA II: - ASA III: 34 (44.7%) ASA IV: 12 (15.8%)	ERCP EUS DBE	376
Present study	200	56.3 ± 15.2	128/72	ASA I: 7 (3.5) ASA II: 73 (36.5) ASA III: 119 (59.5) ASA IV: 1 (0.5)	ERCP	287
**Studies**	**Number of patients with hypoxemia**	**Number of patients with hypotension**	**Number of patients with bradycardia**		**Failure to complete examination**	
Vargo et al. [11]	14 (37%)	6 (15.8%)	NA		NA	
Riphaus et al. [9]	8 (11%)	6 (8%)	3 (4%)		NA	
Kongkam et al. [8]	15 (22.4%)	6 (9%)	2 (3%)		3	
Schilling et al. [10]	9 (11.8%)	4 (5.2%)	5 (6.5%)		NA	
Present study	18 (9%)	18 (9%)	0 (0%)		2 (1%)	
**Studies**	**Procedure time,[min]**	**Endoscopist-reported satisfaction**	**Patient-reported satisfaction**		**Time to recovery, [min]**	
Vargo et al. [11]	53.6 ± 3	8.17 ± 0.28	9.01 ± 0.3		18.6 ± 6.5	
Riphaus et al. [9]	29 ± 19	8.7 ± 1.7	8.4 ± 1.9		22 ± 7	
Kongkam et al. [8]	39.8 ± 32.5	7.4	No significant difference		17.24 ± 5.99	
Schilling et al. [10]	42 ± 18	7 ± 2	NA		NA	
Present study	25 ± 16	9.3 ± 1.3	9.6 ± 0.8		14 ± 10	

Bradycardia: heart rate < 40 bpm; *DBE* Double-balloon enteroscopy, *EUS* Endoscopic ultrasound; Hypotension: SBP < 90 mmHg; Hypoxemia: O_2_ saturation < 90%; NA: data not recorded

The studies included in the Cochrane Review focused on propofol sedation administered by non-anesthesia personnel, and hypoxemia was the most common sedation-related complication (in 11–37% of the patients) [7–11]. Sedation-related hypoxemia occurred in only 9% of our patients. This is an unusual finding, particularly because we used a propofol-remifentanil combination for sedation, which should reduce the propofol dose required, thereby reducing the cardiocirculatory adverse events associated with propofol use, although the risk of respiratory events is higher. Our findings could be attributed to the fact that oxygen supplementation in patients led to safer sedation, but early desaturation was not detected in such cases. Kongkam et al. [8] and Vargo et al. [11] reported higher hypoxemia rates (22.4 and 37%) during sedation administered by non-anesthesia personnel without oxygen supplementation than those reported by studies in which patients received oxygen supplementation (11–11.8%) [9, 10]. With regard to anesthesiologist-administered sedation, Berzin et al. reported a hypoxemia rate of 12.5% and sedation-related adverse events in 21% of cases [17]. Smith et al. also reported a hypoxemia rate of 9.5% and sedation-related adverse events in 19% of cases [18]. These findings concur with those of our study and indicate that sedation administered by anesthesia personnel is safer with regard to hypoxemia.

This result might also be attributed to anesthesiologists’ skills to better manage sedation-related complications such as performing an adequate airway management (e.g. intubation) in case of hypoxemia.

Kongkam et al. reported a procedure termination rate of 4.5% secondary to sedation-related complications (agitation, aspiration, and apnea) with propofol sedation administered by non-anesthesia personnel [8]. In the study reported by Vargo et al., failure to complete the procedure was not recorded [11]. The procedure termination rate was 1% in our study, which was associated with sedation-related hypoxemia secondary to aspiration and apnea. Oxygen saturation returned to normal levels in both patients with minor airway interventions, followed by complete recovery. Most patients in our study (59.5%) were classified as ASA class III, whereas most patients described by Vargo et al. and Kongkam et al. were classified as ASA classes I and II. The mean age of our patients was 56.3 years, which was comparable to the mean age of patients included in the studies reported by Kongkam et al. and Vargo et al. [8, 11]. Buxbaum et al. reported a failure rate of 7% secondary to sedation-related complications in cases of gastroenterologist-administered sedation and 1.3% in cases of sedation administered by anesthesia personnel [19], which concur with our findings. We observed that compared with patients receiving sedation administered by non-anesthesia personnel, those receiving sedation administered by anesthesia personnel showed a better safety profile and lower termination rate.

Hypotension occurred in 5.2–15.8% and bradycardia in 0.0–6.5% of the patients receiving propofol sedation administered by non-anesthesia personnel across studies included in the Cochrane Review [7–11]. Our study results concur with these findings. All patients with hypotension were successfully treated with intravenous fluid replacement, and no procedure was terminated secondary to cardiocirculatory complications. Berzin et al. and Smith et al. reported similar hypotension rates of 7.2–9.5% in patients undergoing anesthesiologist-administered sedation [17, 18].

The cannulation rate was 100% in our study, and the mean procedure time was 25 ± 16 min, which is shorter than that observed in studies reporting sedation administered by non-anesthesia personnel (29–54 min) [8–11], which was attributed to the more rapid and deeper sedation performed by anesthesiologists. Non-anesthesia personnel might be hesitant with regard to drug administration, and endoscopists usually accept suboptimal sedation quality when propofol is administered under their supervision. This hypothesis is supported by the fact that the endoscopist satisfaction rate was higher in procedures performed with sedation administered by anesthesia personnel [15].

The mean recovery time was 14 ± 10 min in our study, and 97% of the patients were awake and oriented upon transfer to the recovery unit. The recovery time was slightly shorter compared with studies reporting sedation administered by non-anesthesia personnel (range 17.2–22 min) [8, 9, 11]. The mean PARS value was 9.5 ± 0.7, which was higher than that reported by Riphaus et al. [9] in a study describing propofol sedation administered by non-anesthesia personnel (mean PARS value 8.3 ± 1.2), indicating more rapid recovery in patients undergoing sedation administered by anesthesia personnel.

Previous studies have reported that compared with patients receiving “conventional sedation” (benzodiazepines alone or combined with opioids), patients receiving propofol sedation for ERCP showed higher post-procedural patient satisfaction [9, 20]. Most patients (mean VAS score: 9.6 ± 0.8) and most endoscopists (mean VAS score: 9.3 ± 1.3) in our study reported being “very satisfied” with the sedation quality. Similar results were reported by Goudra et al. (mean VAS [patients]: 9.8, mean VAS [endoscopists]: 9) in patients receiving sedation administered by anesthesia personnel; however, a significantly higher propofol dosage was used in the study [15]. Notably, the quality of sedation administered by non-anesthesia personnel was associated with low patient and endoscopist satisfaction (mean VAS [patients]: 7.2, mean VAS [endoscopists]: 6) [15]. These results concur with those of other studies describing low patient and endoscopist satisfaction associated with sedation administered by non-anesthesia personnel [8–11]. Compared with non-anesthesia personnel, anesthesiologists usually administer higher propofol doses, thereby achieving deeper sedation and better sedation quality reported by patients and endoscopists [15].

Reportedly, the ERCP-induced complication and mortality rates are 5–10 and 0.1%–1%, respectively [1–5]. The most common post-procedural complication is post-ERCP pancreatitis (prevalence rate 1–15%) [3, 21, 22]. Complication rates in our study were significantly low (4.5%), and post-ERCP pancreatitis occurred in only 1% of patients. The mortality rate was 1%; however, these patients died of non-sedation related events secondary to underlying disease. Lapidus et al. reported high safety and efficacy of endoscopist-administered balanced propofol sedation during ERCP without any adverse outcomes [23]. However, patients included in their study were classified as ASA classes I and II (indicating inclusion of a large percentage of low-risk patients). In concordance with this, the recently updated European Society of Gastrointestinal Endoscopy guidelines suggest “primary involvement of an anesthesiologist” to administer propofol in patients with ASA and/or Mallampati scores ≥3 or in those with comorbidities predisposing to airway obstruction [24].

Limitations of this study included the uncontrolled design, which makes it difficult to compare outcomes of anesthesiologist-administered sedation with those of non-anesthesiologist-administered sedation. Nevertheless, discussed study results and performed comparisons between this study and various other studies might provide an indication in this context. Moreover, despite the large number of patients included in this study our data are only the results of a single-center study and it might not be possible to generalize our findings. Although this study has certain limitations, it does provide a start for future studies evaluating the role of anesthesia personnel in the administration of sedation regarding safety and efficacy for complex endoscopic procedures compared to sedation administered by non-anesthesia personnel. If the results of our study could be confirmed in larger multicenter studies with randomized controlled design, they could have an important impact on guidelines for sedation during complex interventional endoscopy.

## Conclusions

We conclude that sedation administered by anesthesia personnel for ERCP is safe and is associated with a high rate of successful and rapid interventions, as well as short post-anesthesia recovery times and high patient and endoscopist satisfaction.

## Data Availability

The datasets used and analyzed during the current study are available from the corresponding author on reasonable request.

## Abbreviations

ASA: American Society of Anesthesiologists
ECG: Electrocardiography
EGD: Esophagogastroduodenoscopy
ERCP: Endoscopic retrograde cholangiopancreatography
EUS: Endoscopic ultrasound
FDA: US Food and Drug Administration
O_2_ sat: Oxygen saturation
PARS: Post Anesthesia Recovery Score
SBP: Systolic blood pressure
VAS: Visual analogue scale
